# Impediments to Enhancement of CPT-11 Anticancer Activity by *E. coli* Directed Beta-Glucuronidase Therapy

**DOI:** 10.1371/journal.pone.0118028

**Published:** 2015-02-17

**Authors:** Yuan-Ting Hsieh, Kai-Chuan Chen, Chiu-Min Cheng, Tian-Lu Cheng, Mi-Hua Tao, Steve R. Roffler

**Affiliations:** 1 Institute of Microbiology and Immunology, National Yang-Ming University, Taipei, Taiwan; 2 Institute of Biomedical Sciences, Academia Sinica, Taipei, Taiwan; 3 Department of Aquaculture, National Kaohsiung Marine University, Kaohsiung, Taiwan; 4 Department of Biomedical Science and Environmental Biology, Kaohsiung Medical University, Kaohsiung, Taiwan; University of Houston, UNITED STATES

## Abstract

CPT-11 is a camptothecin analog used for the clinical treatment of colorectal adenocarcinoma. CPT-11 is converted into the therapeutic anti-cancer agent SN-38 by liver enzymes and can be further metabolized to a non-toxic glucuronide SN-38G, resulting in low SN-38 but high SN-38G concentrations in the circulation. We previously demonstrated that adenoviral expression of membrane-anchored beta-glucuronidase could promote conversion of SN-38G to SN-38 in tumors and increase the anticancer activity of CPT-11. Here, we identified impediments to effective tumor therapy with *E. coli* that were engineered to constitutively express highly active *E. coli* beta-glucuronidase intracellularly to enhance the anticancer activity of CPT-11. The engineered bacteria, *E. coli* (lux/βG), could hydrolyze SN-38G to SN-38, increased the sensitivity of cultured tumor cells to SN-38G by about 100 fold and selectively accumulated in tumors. However, *E. coli* (lux/βG) did not more effectively increase CPT-11 anticancer activity in human tumor xenografts as compared to non-engineered *E. coli*. SN-38G conversion to SN-38 by *E. coli* (lux/βG) appeared to be limited by slow uptake into bacteria as well as by segregation of *E. coli* in necrotic regions of tumors that may be relatively inaccessible to systemically-administered drug molecules. Studies using a fluorescent glucuronide probe showed that significantly greater glucuronide hydrolysis could be achieved in mice pretreated with *E. coli* (lux/βG) by direct intratumoral injection of the glucuronide probe or by intratumoral lysis of bacteria to release intracellular beta-glucuronidase. Our study suggests that the distribution of beta-glucuronidase, and possibly other therapeutic proteins, in the tumor microenvironment might be an important barrier for effective bacterial-based tumor therapy. Expression of secreted therapeutic proteins or induction of therapeutic protein release from bacteria might therefore be a promising strategy to enhance anti-tumor activity.

## Introduction

Specific delivery of therapeutic enzymes to cancer cells for subsequent activation of anticancer drugs in the tumor microenvironment is a promising approach to improve the selectivity of cancer chemotherapy [1,2]. Different vehicles including viruses [3,4], liposomes [5] and antibodies [6,7] have been evaluated to deliver therapeutic enzymes to cancer cells. Recently, bacteria directed enzyme prodrug therapy (BDEPT) has been investigated for cancer therapy [8]. In this approach, engineered bacteria are employed to deliver therapeutic enzymes to tumors. Although the mechanism of tumor localization is still unclear, many bacterial species have been found to selectively localize and proliferate in tumors after systemic administration, including *Clostridium* [9,10], *Bifidobacterium* [11,12], *Salmonella* [13,14] and *E. coli* [15,16]. Using bacteria as a delivery vehicle possesses potential advantages as compared to other delivery systems. Bacteria can proliferate in tumors for several weeks [17,18], thereby providing sustained expression of therapeutic enzymes to facilitate multiple rounds of drug therapy. Bacteria tumor targeting might also represent a universal treatment method since tumor colonization seems to be independent of the type of cancer being treated [15,19,20]. Bacteria colonization of tumors is highly specific and can achieve much greater tumor/normal tissue ratios than other targeted therapies such as antibodies [21–24] and nanoparticles [25–28]. This can help decrease off-target toxicity of anticancer drug therapy.

**Fig 1:**
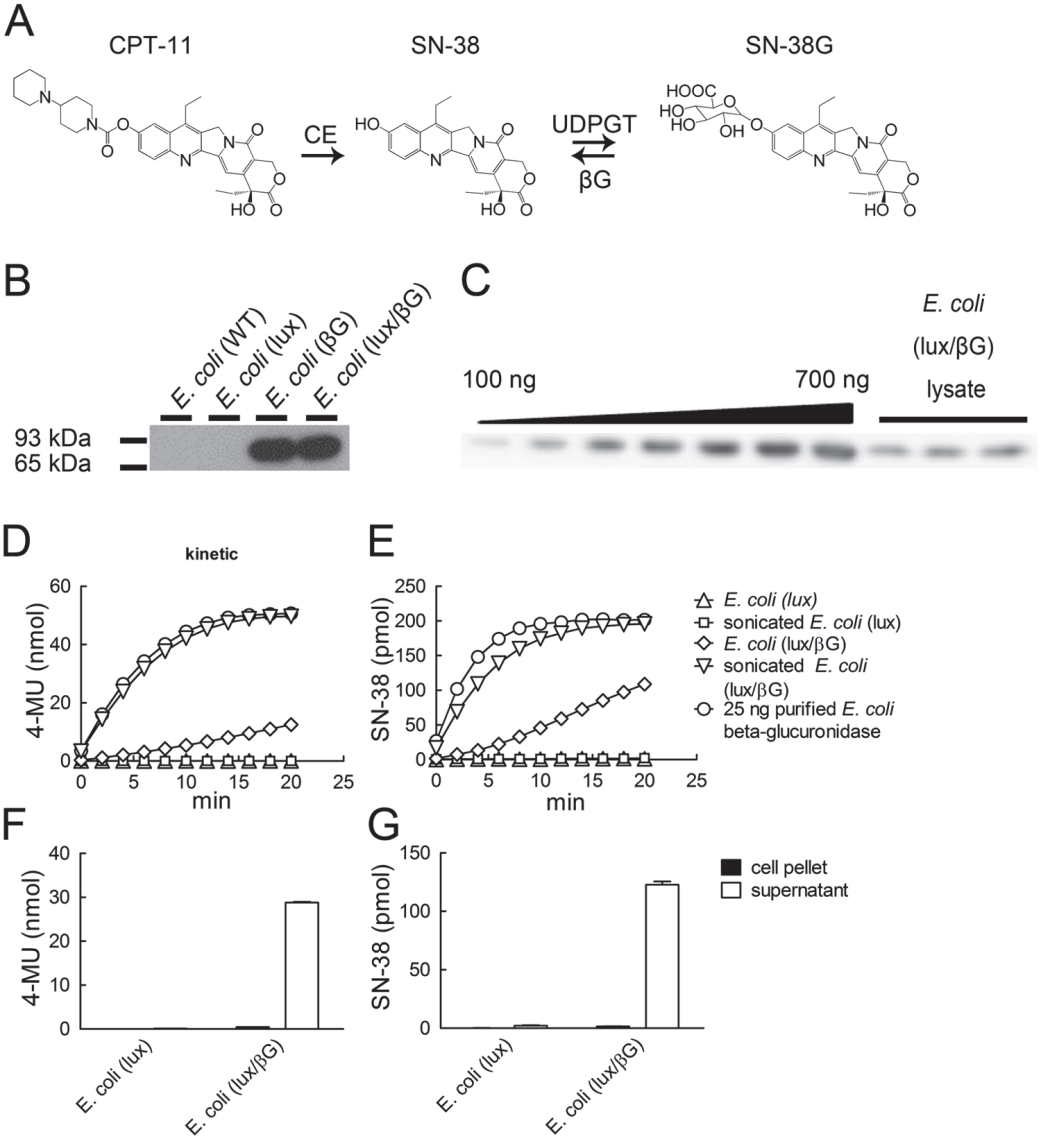
Beta-glucuronidase expression and activity of transformed bacteria. (A) CPT-11 metabolism. CPT-11 can be converted to SN-38 by carboxylesterase (CE). SN-38 can be further metabolized to SN-38G by UDP-glucuronosyltransferase (UDPGT). SN-38G can be reconverted by beta-glucuronidase (βG) to SN-38. (B) Lysates prepared from 4 x 10^6^ c.f.u. *E. coli* were immunoblotted with mouse anti-*E. coli* beta-glucuronidase (1E8) monoclonal antibody and goat anti—mouse IgG-HRP. The chemiluminescence signal was detected by X-ray film. (C) Defined amounts of recombinant *E. coli* beta-glucuronidase or lysates prepared from 2 x 10^7^ c.f.u. *E. coli* (lux/βG) were immunoblotted as above. The chemiluminescence signal was detected by Fuji LAS-3000. (D) 500 μM 4-MUG or (E) 2 μM SN-38G was incubated with 25 ng purified recombinant. *E. coli* beta-glucuronidase, 2 x 10^6^ c.f.u. *E. coli* (lux), 2 x 10^6^ c.f.u. *E. coli* (lux/βG) or lysates prepared form the same number of bacteria. The formation of 4-MU and SN-38 at each time point was measured as described in materials and methods (n = 3). 2 x 10^6^ c.f.u. *E. coli* were mixed with (F) 500 μM 4-MUG or (G) 2 μM SN-38G at 37°C for 30 min and then centrifuged to separate the supernatant and pellet. Fluorescence was detected and analyzed (n = 3).

CPT-11 (Irinotecan) is currently used as a single agent or in combination with 5-FU/leucovorin for the chemotherapy of colorectal adenocarcinoma [29]. CPT-11 is hydrolyzed by carboxylesterases into SN-38, which is believed to be the active form of the drug responsible for the anticancer activity of CPT-11 [30] (Fig. 1A). SN-38 is a topoisomerase I poison that causes the formation of a stable complex between DNA, topoisomerase I and SN-38, resulting in stabilization of single-strand DNA breaks and cancer cell apoptosis [31,32]. However, SN-38 is rapidly metabolized to the non-toxic and largely inactive glucuronide conjugate SN-38G by UDP glucuronosyltransferase (UDPGT) in the liver [33]. The concentration of SN-38G is up to 10-fold higher than SN-38 in the plasma of patients receiving CPT-11 [34,35], and therefore represents a potential target for enzyme-mediated prodrug therapy. Indeed, we previously showed that expression of a membrane-anchored form of murine beta-glucuronidase in tumors can enhance CPT-11 anti-tumor efficacy by conversion of SN-38G into SN-38 in the tumor microenvironment [4,36,37].

Here, we investigated if the anticancer activity of CPT-11 could be improved by systematic administration of *E. coli* that was engineered to express *E. coli* beta-glucuronidase. *E. coli* were selected for tumor colonization because they proliferate less in normal tissues compared to other strains of bacteria [18,19,38]. We anticipated that tumor-located expression of *E. coli* beta-glucuronidase would more effectively convert SN-38G to SN-38 as compared to viral-mediated expression of murine beta-glucuronidase in tumors [4] since the bacterial enzyme displays about one hundred fold greater catalytic activity as compared to murine beta-glucuronidase [36]. We found that *E. coli* engineered to over express beta-glucuronidase can convert SN-38G to SN-38 and enhance the anticancer activity of SN-38G to cultured cancer cells by about 100 fold. *In vivo* biodistrubution studies demonstrated that *E. coli* specifically colonized tumors in both immunodeficient and immune competent mice. However, in contrast to adenovirus-mediated expression of murine beta-glucuronidase in tumors, microbially-delivered *E. coli* beta-glucuronidase did not significantly enhance the antitumor activity of CPT-11. Slow uptake of glucuronides into *E. coli* coupled with the preferential accumulation of *E. coli* in necrotic regions of tumors appears to hinder efficient activation of systemically-administered glucuronide drugs, leading to poor *in vivo* antitumor activity. Our results suggest that secreted enzymes might be more effective for BDEPT approaches to cancer therapy.

## Materials and Methods

### Bacteria and plasmids

*E. coli* DH5α (F- φ80*lac*ZΔM15 Δ(*lac*ZYA-*arg*F) U169 *rec*A1 *end*A1 *hsd*R17 (r_k_-, m_k_+) *pho*A *sup*E44 λ- *thi*-1 *gyr*A96 *rel*A1) was from Bioman Scientific (Taipei, Taiwan). The plasmid constructs have been described [15]. Briefly, to constitutively express beta-glucuronidase or luciferase, we first replaced the T7 promoter in the pRSETB vector (Invitrogen, Grand Island, NY) with the fen promoter from pGHL6 [39] to produce pRSETB-fen. The *E. coli* beta-glucuronidase gene was PCR amplified from genomic DNA isolated from *E. coli and* cloned into pRESTB-fen to generate pRESTB-fen-βG. The luxCDABE gene cluster (generously provided by Dr. EA Meighen, Department of Biochemistry, McGill University, Montreal, Quebec, Canada) [39] was cloned into pRSETB-fen to create pRSETB-fen-lux. The fen-βG fragment was also cloned into pRSETB-fen-lux to form pRSETB-lux/βG. These three plasmids were individually transformed into *E. coli* DH5alpha to constitutively produce *E. coli* beta-glucuronidase, luciferase or both gene products, respectively.

### Reagents

RPMI-1640, MEM-α, and fluorescein di-β-D-glucuronide (FDGlcU) were from Invitrogen (Grand Island, NY). Bovine calf serum (BCS) was from HyClone (Logan, Utah). 4-methylumbelliferyl-β-D-glucuronide (4-MUG), CPT-11, lysozyme, and DNase I were from Sigma-Aldrich (Saint Louis, MO). SN-38G was purified by HPLC from urine of CPT-11-treated mice [40]. SN-38 was from Sinopharm (Beijing, China). ^3^H-thymidine was from PerkinElmer, Inc. (Boston, MA).

### Animals and cells

6 to 8-week-old NOD.CB17-Prkdcscid/IcrCrlBltw mice were purchased from BioLASCO Taiwan Co., Ltd (Taipei, Taiwan). Animal experiments were performed under specific pathogen free conditions in accordance with institute guidelines. Protocols were approved by Academia Sinica Institutional Animal Care & Utilization Committee (Permit Number: 12–12–451). HCT116 (ATCC No. CCL-247) and LS174T (ATCC No. CL-188) human colorectal carcinoma cells were from the American Type Culture Collection (Manassas, VA). CL1–5 human lung adenocarcinoma cells were a gift from Dr. Pan-Chyr Yang (National Taiwan University, Taipei, Taiwan) [41]. HCT116 cells and CL1–5 cells were cultured in RPMI-1640 medium containing 2.98 mg/ml HEPES, 1 mg/ml sodium bicarbonate, and 10% BCS. LS174T cells were cultured in MEM-α medium containing 1 mg/ml sodium bicarbonate and 10% BCS. All cells were cultured in a humidified atmosphere of 5% CO_2_ in air at 37°C.

### Western blotting and quantification of beta-glucuronidase expression

**Fig 2:**
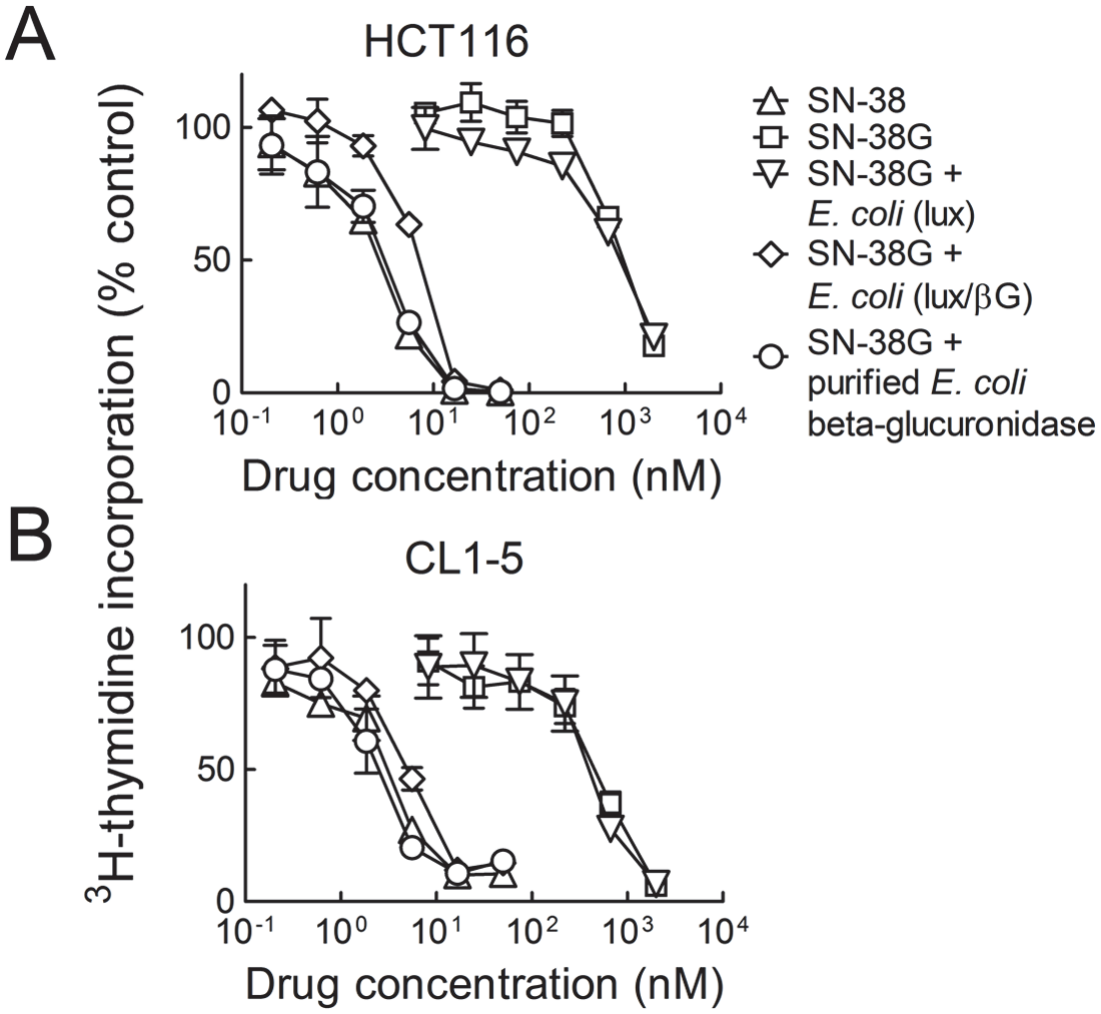
Cytotoxicity of bacterially-activated SN-38G. Defined concentrations of SN-38G were reacted with 1 μg recombinant *E. coli* beta-glucuronidase or 2 x 10^6^ c.f.u. *E. coli* (lux) or *E. coli* (lux/βG) at 37°C for 2 h. The bacteria were removed by filtration and serial dilutions were then incubated with HCT116 human colorectal carcinoma cells (A) or CL1–5 human lung adenocarcinoma cells (B) for 48 h. HCT116 and CL1–5 cells were also treated with serial dilutions of SN-38 and SN-38G as controls. The cells were incubated with fresh medium for an additional 24 h before incorporation of ^3^H-thymidine into cellular DNA was measured. n = 3. Bars, s.d.

0.5 ml (Fig. 1B) or 2 ml (Fig. 1C) *E. coli* (lux/βG) (OD_600_ = 0.5, ~8 x 10^7^ c.f.u./ml) and defined amounts of purified recombinant *E. coli* beta-glucuronidase [42] were separated on a 10% SDS-PAGE gel and transferred to nitrocellulose paper. The membranes were incubated with 0.6 μg/ml mouse anti-*E. coli* beta-glucuronidase antibody 1E8 [43] followed by goat anti-mouse IgG Fc-HRP (Organon Teknika Corporation, Durham, NY). Antibody binding was detected by SuperSignal West Pico Chemiluminescence Substrate (Thermo Scientific, Rockford, IL). The luminescence was visualized on an X-ray film (Fig. 1B) or in a Fuji LAS-3000 imager (Fig. 1C) to quantify the protein amounts using Metamorph software (Molecular Devices, Sunnyvale, CA).

### *E. coli* beta-glucuronidase activity

1 ml of *E. coli* (OD_600_ = 0.5) was washed with PBS and suspended in 1 ml reaction buffer (50 mM bis-Tris, 50 mM triethanolamine, 100 mM acetic acid, 0.1% bovine serum albumin, pH 7). To break the cell wall, 1 ml of *E. coli* (OD_600_ = 0.5) was washed with reaction buffer and then suspended in reaction buffer containing 0.2 mg/ml lysozyme and DNase I at 37°C for 15 min followed by sonication for 15 min on ice. 50 μl live *E. coli* or *E. coli* lysate was incubated with 50 μl 4-MUG (500 μM) or SN-38G (4 μM) in 96-well black microtiter plates at 37°C. Relative fluorescence was measured with a fluorescence microplate reader (Molecular Devices) and the 4-MU formation was calculated from a standard curve. The excitation and emission wavelengths of 4-MU, SN-38 and SN-38G were 355/460, 375/470, and 375/560, respectively. Fluorescence was detected every 2 minutes for 20 minutes. The SN-38 concentration was calculated by:

$$A_{470}=\varepsilon_{\text{470SN-38}}\text{* }C_{\text{SN-38}}\text{ + }\varepsilon_{\text{470SN-38G}}\text{* }C_{\text{SN-38G}}$$

$$A_{560}=\varepsilon_{\text{560SN-38}}\text{* }C_{\text{SN-38}}\text{ + }\varepsilon_{\text{560SN-38G}}\text{* }C_{\text{SN-38G}}$$

where A is the absorbance, ε is the absorption coefficient and C is the concentration of each molecule.

To determine whether the hydrolyzed substrates were released from the bacteria, 50 μl live *E. coli* were incubated with 50 μl 4-MUG (500 μM) or SN-38G (4 μM) at 37°C for 5 minutes and then the bacteria were pelleted at 5000xg for 5 minutes. The fluorescence of the cell pellet and supernatant were measured and converted into 4-MU and SN-38 concentrations. To detect beta-glucuronidase activity of *E. coli* in tumor tissues, tumors were isolated from mice and then homogenized in PBS on ice. 50 μl tissue lysates were reacted with 50 μl 4-MUG (500 μM) at 37°C for 30 minutes and the 4-MU formation was calculated.

### Recombinant *E. coli* and mouse beta-glucuronidase specific activity

Recombinant *E. coli* and mouse beta-glucuronidase were produced as described [44]. 1 ng *E. coli* beta-glucuronidase and 10 ng mouse beta-glucuronidase were incubated with 2 μM SN-38G at 37°C. The SN-38 formation at pH 7 was measured at 0, 5, 10, 15, 20 and 30 minutes (n = 3). One unit beta-glucuronidase activity corresponds to the hydrolysis of 1 pmole SN-38G per h at 37°C.

### Luciferase activity

*E. coli* (50 μl, OD_600_ = 0.5) suspended in PBS were added into 96-well white plates and luminescence was detected on a Top Count Luminescence Counter (Perkin-Elmer Life Sciences, Waltham, MA).

### ^3^H-thymidine incorporation assay

5000 HCT116 or CL1–5 cells per well were seeded in 96-well cell culture plates for 8 h. 1.5 x 10^7^ c.f.u. *E. coli* (lux) or *E. coli* (lux/βG) were mixed with 1.5 ml 2 μM or 50 nM SN-38G at 37°C for 2 h and then the bacteria were removed by filtration through a 0.2 μm filter. SN-38, SN-38G or *E. coli* treated SN-38G were 3 fold serially-diluted in culture medium and then added to HCT116 or CL1–5 cells for 48 h. The culture medium was removed and fresh culture medium containing ^3^H-thymidine (1 μCi/well) was added for another 16 h. Radiation incorporated into cellular DNA was then measured on a Top Count scintillation counter. The results are expressed as ^3^H-thymidine incorporation (% control) = (c.p.m._(S)_ x 100)/c.p.m._(C)_ where c.p.m. represents counts per minute of sample (S) or untreated controls (C).

### Adenovirus preparation

The pAd-CMV plasmids containing membrane-tethered mouse beta-glucuronidase or anti-dansyl single-chain antibody transgenes [45] were co-transfected with pJM17, which carries the entire Ad5 genome, lacking E1 and E3 functions, in E1-complementing 293N cells to produce adenoviruses expressing membrane tethered mouse beta-glucuronidase (Ad/mβG) or anti-dansyl single-chain antibody (Ad/DNS). Virus production, purification and titer determination were as previously described [4].

### *In vivo* imaging

NOD/SCID mice were s.c. inoculated with 10^7^ CT26 mouse colon cancer cells, EJ human bladder cancer cells, HCT116 human colon cancer cells, or 2 x 10^6^ LS174T human colon cancer cells on the right flank. BALB/c mice were s.c. inoculated with 10^7^ CT26 mouse colon cancer cells. 4 x 10^7^ c.f.u. *E. coli* were i.v. injected in each mouse when the tumor volumes reached ~150 mm^3^. The relative body weight was measured every 2 or 3 days. The localization of *E. coli* in mice was detected by luminescence emission. Under gas anesthesia, the luminescence in tumors was detected daily on an IVIS Spectrum (Caliper Lifesciences, Hopkinton, MA). The region of interest (ROI) was analyzed with Living Image Software (Caliper lifesciences). To measure the *in vivo* enzyme activity, mice were either intratumorally-injected on two consecutive days with 10^9^ p.f.u. Ad/mβG or Ad/DNS when tumor volumes reached ~100 mm^3^ or i.v. injected with 4 x 10^7^ c.f.u. *E. coli* (lux) or *E. coli* (lux/βG) when the tumor volume reached ~150 mm^3^. On the fourth day after administration of *E. coli* or adenovirus, mice received 500 μg FDGlcU by intravenous injection or 5 μg FDGlcU by direct intratumor injection, respectively. The fluorescence in tumors was detected on an IVIS Spectrum 30 minutes later. To investigate the effect of releasing beta-glucuronidase from *E. coli*, *E. coli* (lux/βG) treated mice were i.v. injected with 500 μg FDGlcU to determine baseline beta-glucuronidase imaging. Two days later, each mouse was treated with 50 μl 4 mg/ml lysozyme and DNase I via intratumor injection to lyse the bacteria and then i.v. immediately injected with 500 μg FDGlcU. After 30 minutes, both control and treated mice were imaged on an IVIS Spectrum for luminescence and fluorescence analysis.

### *Ex vivo E. coli* counting, imaging and tissue section staining

To examine the biodistribution of *E. coli* in mice, tumors and other normal organs were removed and imaged on an IVIS Spectrum 8 days after i.v. administration of 4 x 10^7^ c.f.u. *E. coli* (lux) or *E. coli* (lux/βG). Samples were homogenized and spread on carbenicillin-containing LB plates to count bacterial colonies. Spleen weights from each group were measured. For immunofluorescence histology, tumor samples were fixed with 4% paraformaldehyde in PBS and embedded with paraffin. Sections were stained with goat anti-*E. coli* polyclonal antibody (Abcam, Cambridge, MA) and biotinylated 1E8 (anti-*E. coli* beta-glucuronidase) followed by rhodamine-conjugated rabbit anti-goat IgG (Organon Teknika Corporation, West Chester, PA) and Alexa Fluor 488 conjugated streptavidin (Invitrogen), respectively. Sections were incubated with 1 μg/ml DAPI to visualize nuclei.

### *In vivo* anti-tumor activity

6 to 8 week-old NOD/SCID mice were s.c. injected on the right flank with 10^7^ HCT116 or LS174T colon cancer cells. The mice were divided into 5 groups (n = 6) and treated with PBS, CPT-11, *E. coli* (lux) and CPT-11, *E. coli* (lux/βG) and PBS, or *E. coli* (lux/βG) and CPT-11. We injected 4 x 10^7^ c.f.u. *E. coli* (lux) or *E. coli* (lux/βG) by a single i.v. injection when tumors reached a mean size of 150 mm^3^. Four days later, mice were i.v. injected with 10 mg/kg CPT-11 or vehicle for two consecutive days. Tumor volumes were calculated according to the formula: length x width x height x 0.5.

### Statistical significance

Statistical significance of differences between mean values was estimated with Excel (Microsoft, Redmond, WA, USA) using the independent t-test for unequal variances. For *in vivo* enzyme releasing image, the ROI value differences were analyzed by GraphPad Prism Version 5 paired t-test. P-values of < 0.05 were considered statistically significant.

## Results

### Characterization of beta-glucuronidase expression and activity of transformed *E. coli*

*E. coli* that constitutively display luminescence and/or express *E. coli* beta-glucuronidase were generated as previously described [15]. Beta-glucuronidase expression in *E. coli* is inducible and normally low unless the bacteria are cultured in the presence of high concentrations of glucuronide carbon sources [46]. Western blot analysis of transformed bacteria showed that beta-glucuronidase was detected in *E. coli* (βG) and *E. coli* (lux/βG) but not in wild-type *E. coli* or *E. coli* (lux) (Fig. 1B). By comparing the levels of beta-glucuronidase in *E. coli* (lux/βG) with purified *E. coli* beta-glucuronidase protein, we estimated that *E. coli* (lux/βG) expressed about 60 ng beta-glucuronidase/10^7^ c.f.u. (Fig. 1C). We assessed if *E. coli* (lux/βG) can hydrolyze glucuronides by adding a synthetic glucuronide substrate (4-MUG) to live bacteria and then measuring the fluorescence of the hydrolyzed product (4-MU). *E. coli* (lux/βG) converted 4-MUG to 4-MU whereas *E. coli* (lux) did not produce measurable 4-MU (Fig. 1D), indicating that ectopically-produced beta-glucuronidase in *E. coli* was active. However, 4-MUG was hydrolyzed much faster by a similar amount of recombinant beta-glucuronidase (25 ng), suggesting that 4-MUG hydrolysis in *E. coli* (lux/βG) may be limited by transport of 4-MUG into *E. coli*. In support of this hypothesis, the rate of 4-MUG hydrolysis in lysates prepared from the same number of *E. coli* (lux/βG) was similar to the rate of 4-MUG hydrolysis by recombinant beta-glucuronidase (Fig. 1D). By contrast, lysates prepared from *E. coli* (lux) did not hydrolyze 4-MUG, verifying that overexpression of beta-glucuronidase was required for glucuronide hydrolysis. Similar results were found for hydrolysis of SN-38G by *E. coli* (lux/βG) (Fig. 1E). Since the substrates were hydrolyzed inside *E. coli*, we examined if 4-MU and SN-38 were released from the bacteria by mixing 4-MUG or SN-38G with *E. coli* and then measuring 4-MU and SN-38 in the medium or cell pellet. 4-MU (Fig. 1F) and SN-38 (Fig. 1G) were mostly found in the supernatant of *E. coli* (lux/βG), indicating that the reaction products were efficiently released from *E. coli*. We conclude that *E. coli* (lux/βG) can uptake 4-MUG and SN-38G and convert and subsequently release 4-MU and SN-38 into the culture medium. However, uptake of the glucuronides into *E. coli* appears to be a rate-limiting step in the conversion process.

### *E. coli* (lux/βG) can increase the cytotoxicity of SN-38G

We investigated if cytotoxic concentrations of SN-38 could be generated from SN-38G by *E. coli* (lux/βG). SN-38 was about 355 and 150 fold more toxic than SN-38G to HCT116 human colon cancer cells (Fig. 2A) and CL1–5 human lung cancer cells (Fig. 2B), respectively. Preincubation of SN-38G with *E. coli* (lux) slightly increased the inhibition of HCT116 and CL1–5 cell growth by 1.08 and 1.15 fold, respectively (Table 1). By contrast, incubation of SN-38G with *E. coli* (lux/βG) increased inhibition of HCT116 and CL1–5 cell growth by 135 and 90 fold, respectively. We conclude that *E. coli* (lux/βG) can generate cytotoxic concentrations of SN-38 from SN-38G.

**Table 1:** *E. coli* (lux/βG) sensitization of cancer cells to SN-38G.

Cells	SN-38	SN-38G	SN-38G + 1 μg *E. coli* βG	SN-38G + *E. coli* (lux)	SN-38G + *E. coli* (lux/βG)
HCT116	2.7 ± 0.1	960 ± 9.1	3.0 ± 0.3	890 ± 21.1	7.1 ± 0.3
CL1–5	3.0 ± 0.3	455 ± 36.2	2.4 ± 0.6	390 ± 57.4	5.0 ± 0.6

Results show the mean IC_50_ values in nM of triplicate determinations ± standard deviation.

### *E. coli* (lux/βG) can specifically accumulate in tumors

**Fig 3:**
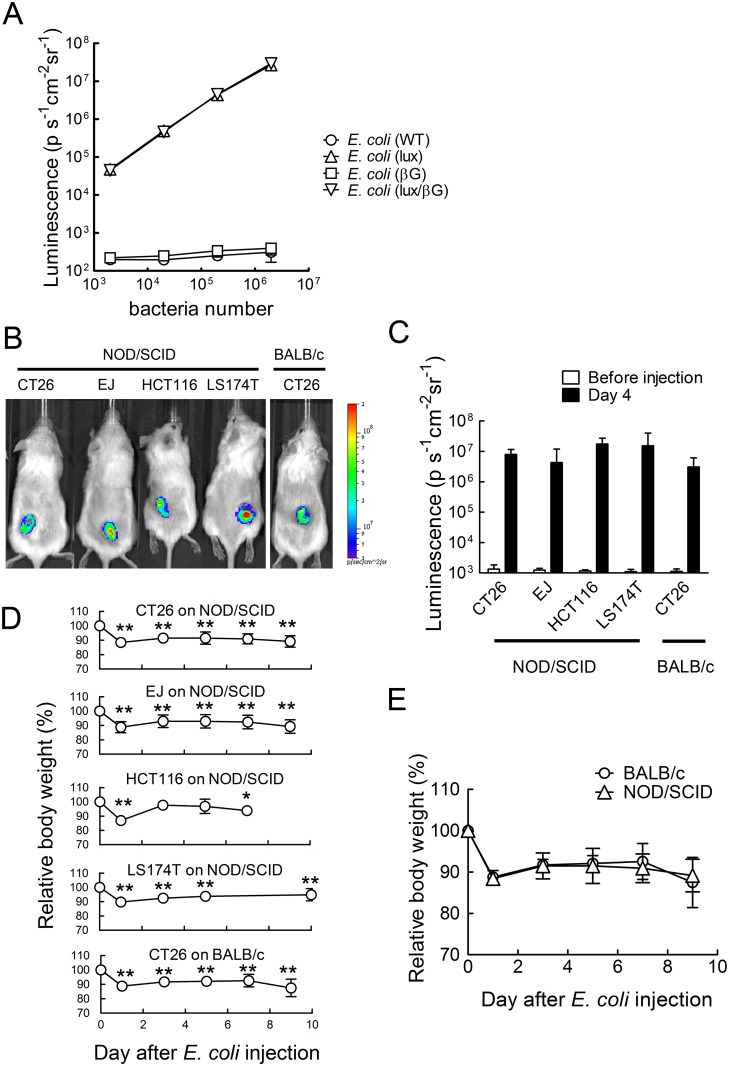
Localization of engineered *E. coli* in tumors. (A) The luminescence of 10 fold serial dilutions of the indicated wild-type or engineered *E. coli* was detected on a Top Count scintillation counter (n = 3). (B) NOD/SCID mice bearing established CT26 mouse colon, EJ human bladder, HCT116 human colon or LS174T human colon tumors and BALB/c mice bearing CT26 colon tumors were i.v. injected with 4 x 10^7^ c.f.u. *E. coli* (lux/βG). Mice were imaged on an IVIS Spectrum before *E. coli* injection and on day 4 to trace the bacteria location. (C) Mean luminescence values of tumors is shown. The Y-axis indicates the luminescence intensity. N = 5. Bars, s.d. (D) Relative body weights of mice are indicated (n = 6). Bars, s.d. * p < 0.05, ** p < 0.005. (E) Comparison of relative body weight loss of BALB/c and NOD/SCID mice bearing established CT26 mouse colon tumors after i.v. injection of 4 x 10^7^ c.f.u. *E. coli* (lux/βG). N = 5. Bars, s.d.

Both *E. coli* (lux) and *E. coli* (lux/βG) can emit luminescence *in vitro* (Fig. 3A) and *in vivo* (Fig. 3B) to facilitate tracing their locations in mice. Imaging of tumor-bearing mice after i.v. injection of *E. coli* (lux/βG) demonstrated specific localization of *E. coli* in tumors that was relatively independent of the tumor type and immune status of the mice (Fig. 3B and 3C). Systemic administration of *E. coli* caused about 12% weight loss to mice on day 1 (Fig. 3D). Mice body weight partially rebounded but remained less than untreated mice. Although most experiments were performed in immune deficient mice to allow investigation of therapy in human xenograft models, we also examined the toxicity of systemic *E. coli* administration to immune competent BALB/c mice. *E. coli* in immune-competent mice retained the ability to colonize tumors (Fig. 3B and 3C). Interestingly, body weight loss was not significantly different in immune deficient and immune competent mice injected with *E. coli* (Fig. 3E), suggesting that *E. coli* (DH5±) do not cause excessive immune-related toxicity.

**Fig 4:**
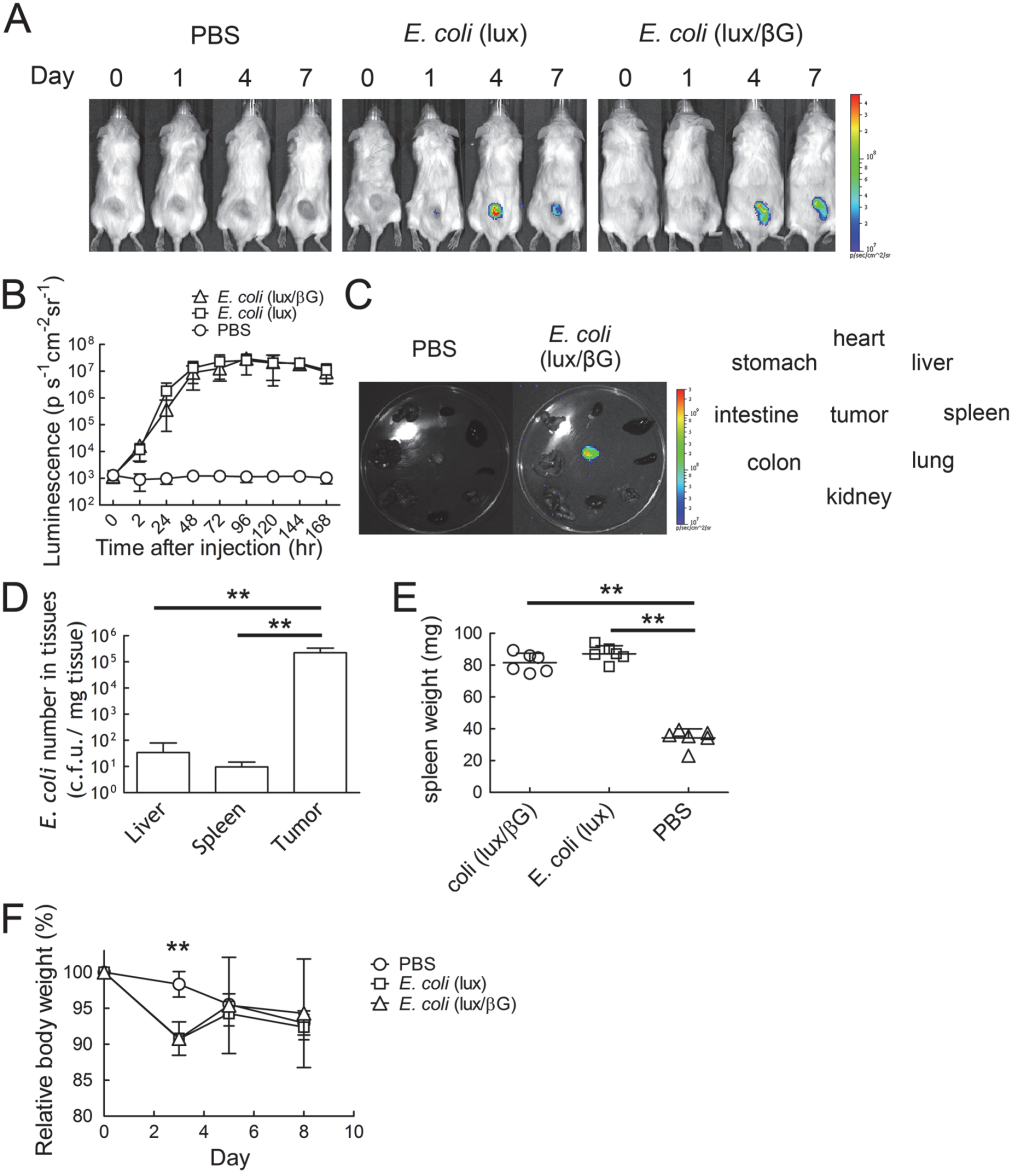
*In vivo* localization of *E. coli* in HCT116 tumors. PBS or 4 x 10^7^ c.f.u. *E. coli* (lux) or *E. coli* (lux/βG) were i.v. injected into mice bearing 150 mm^3^ HCT116 tumors. (A) Luminescence images were captured on an IVIS Spectrum on days 0, 1, 4 and 7 (n = 6). (B) The region of the interesting (ROI) of mice treated as in panel A on different days were analyzed (n = 6). The Y-axis indicates the luminescence intensity. Bars, s.d. (C) The luminescence of organs (clockwise order: heart, liver, spleen, lung, kidney, intestine, colon, and stomach) and tumors isolated from mice treated with PBS or 4 x 10^7^ c.f.u. *E. coli* (lux/βG) were imaged on an IVIS spectrum on day 8. Bars, s.d. ** P < 0.005. (D) *E. coli* (lux/βG)-injected mice were sacrificed on day 8. Liver, spleen and tumor tissues were homogenized and spread on LB agar plates to count the bacterial colonies (n = 6). Bars, s.d. ** P < 0.005. (E) The spleen weight on day 8 and (F) relative body weight of mice during the experiment were measured (n = 6). Bars, s.d. ** P < 0.005.

The time course of *E. coli* accumulation in ~150 mm^3^ HCT116 tumors in NOD/SCID mice was examined after i.v. injection of 4x10^7^ c.f.u. *E. coli*. Luminescence could be detected in tumors within 24 hour [4/6 for *E. coli* (lux), 3/6 for *E. coli* (lux/βG)] (Fig. 4A). The luminescence of *E. coli* (lux) and *E. coli* (lux/βG) in tumors reached a plateau from days 3 to 7 (Fig. 4B), demonstrating that *E. coli* localized and proliferated in tumors. Imaging of isolated tumors and normal tissues taken from mice on day 8 showed strong luminescence in tumors but not in normal tissues (Fig. 4C). Determination of the numbers of *E. coli* in different organs also showed that tumors accumulated about 6,500 and 23,000 fold higher levels of *E. coli* than did the liver and spleen, respectively (Fig. 4D). Mice injected with *E. coli* (lux) or *E. coli* (lux/βG) displayed splenomegaly (Fig. 4E), suggesting that the systemically-administered *E. coli* induced an immune response. This immune response may cause the transient body weight loss (9.2%) on day 3 observed after systematic administration of *E. coli* (Fig. 4F).

### *E. coli* accumulates in necrotic areas of tumors

**Fig 5:**
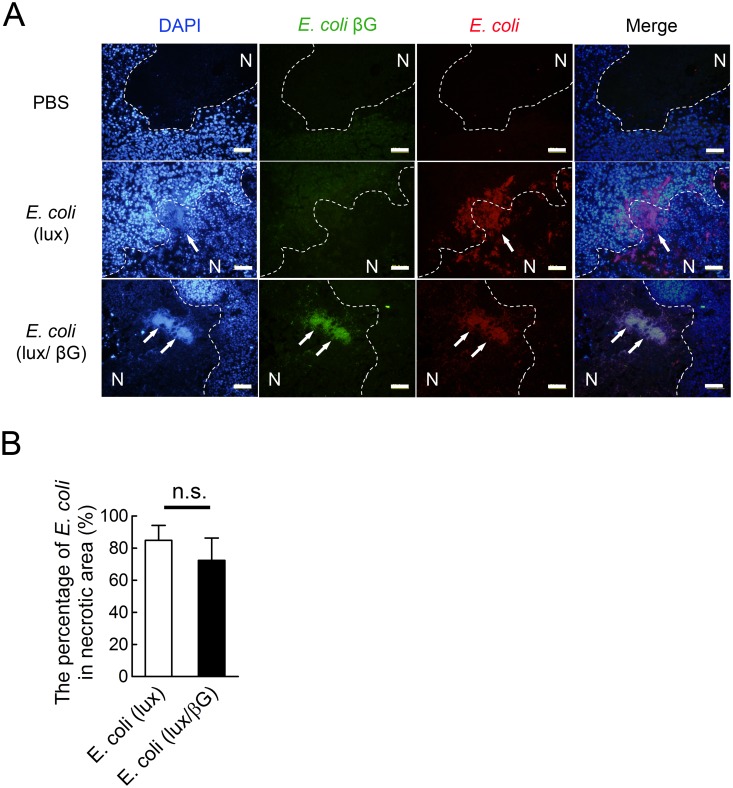
The distribution of *E. coli* in tumors. (A)Tumor sections obtained on day 8 from mice injected with PBS, *E. coli* (lux/βG) or *E. coli* (lux) were stained with DAPI (blue), mouse anti-*E. coli* beta-glucuronidase antibody (green) and goat anti-*E. coli* antibody (red). Arrows indicate the location of *E. coli*. Dotted lines indicate the approximate border between necrotic areas and live cells. N, necrotic area. Bars, 50 μm. (B) The percentage of *E. coli* in the necrotic area of tumor tissue sections. n = 4. n.s., no significant difference.

The distribution of bacteria in HCT116 tumors was examined in more detail by immunohistochemical analysis of *E. coli* and beta-glucuronidase in tumor sections from *E. coli* (lux/βG), *E. coli* (lux), or PBS-treated mice. DAPI (blue) stained DNA in both live tumor cells and *E. coli*. DAPI-stained *E. coli* were observed as tiny spots that formed a dense cluster under UV excitation (Fig. 5A). Antibody staining for *E. coli* further confirmed the location of the bacteria. Anti-*E. coli* beta-glucuronidase antibody staining showed that *E. coli* (lux/βG) but not *E. coli* (lux) expressed beta-glucuronidase in the tumor microenvironment. 72.4% ±13.9% of *E. coli* (lux) and 84.9% ± 9.2% of *E. coli* (lux/βG) were located in necrotic areas of tumors as distinguished by DAPI staining. *E. coli* (lux) and *E. coli* (lux/βG) didn’t show significant differences in localization to tumor necrotic areas (Fig. 5B). The bacteria distribution indicates that *E. coli* primarily accumulated at the border between viable and necrotic cells.

### Anti-tumor activity of *E. coli* (lux/βG) and CPT-11 *in vivo*

**Fig 6:**
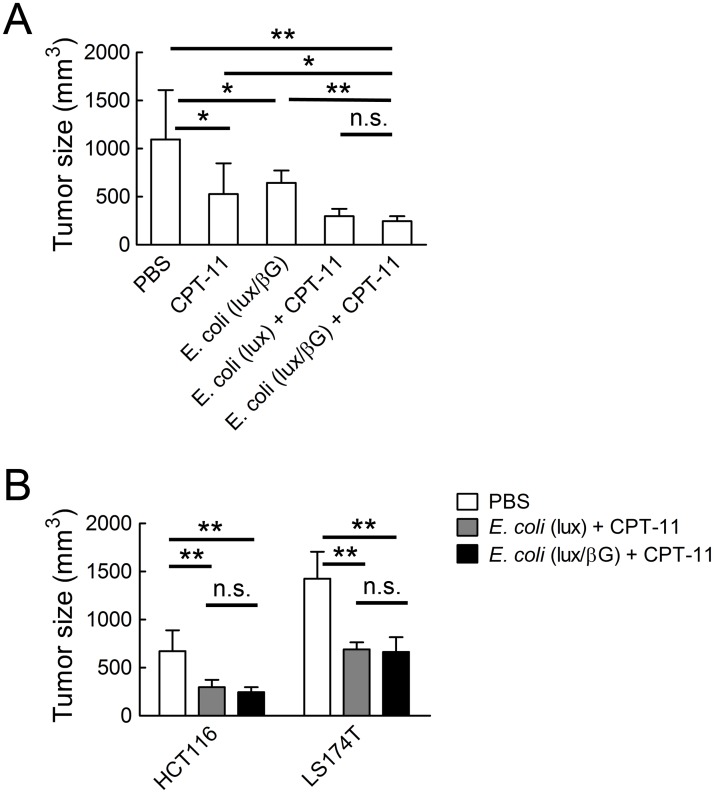
Anti-tumor activity of CPT-11 and *E. coli* (lux/βG). (A) 4 x 10^7^ c.f.u. *E. coli* (lux) or *E. coli* (lux/βG) were i.v. injected into NOD/SCID mice bearing ~150 mm^3^ HCT116 tumors. After 4 days, 10 mg/kg CPT-11 or vehicle (PBS) were i.v. injected on two consecutive days. Tumor sizes were measured 21 days after treatment. Results show mean values of 7 mice. Bars, s.d. (B) NOD/SCID mice bearing established HCT116 (n = 7) or LS174T tumors (n = 6) were i.v. injected with PBS, or 4 x 10^7^ c.f.u. *E. coli* (lux) or *E. coli* (lux/βG). All the mice were treated with 10 mg/kg CPT-11 on days 4 and 5. Tumor sizes were measured on day 10. Bars, s.d. * P < 0.05; ** P < 0.005; n.s. no significant difference.

To determine whether *E. coli* (lux/βG) could enhance the anti-tumor activity of CPT-11, NOD/SCID mice bearing established HCT116 human colon tumors were i.v. injected with PBS or *E. coli*. Four days later, the mice were i.v. injected with 10 mg/kg CPT-11 or vehicle on two consecutive days. Treatment of mice with either CPT-11 or *E. coli* (lux/βG) alone significantly suppressed tumor growth as compared to treatment with vehicle alone (Fig. 6A). Combination treatment with CPT-11 and *E. coli* (lux/βG) produced significantly greater antitumor activity that either individual treatment. However, this effect did not depend on bacterial expression of beta-glucuronidase because treatment of mice with CPT-11 and *E. coli* (lux) suppressed tumor growth to a similar extent (Fig. 6A). Similar results were found for LS174T human colorectal tumors in NOD/SCID mice; treatment with CPT-11 and either *E. coli* (lux/βG) or *E. coli* (lux) produced significant but similar suppression of tumor growth (Fig. 6B), indicating that therapeutic outcome was not affected by expression of beta-glucuronidase in *E. coli* (lux/βG).

### *In vivo* beta-glucuronidase activity

**Fig 7:**
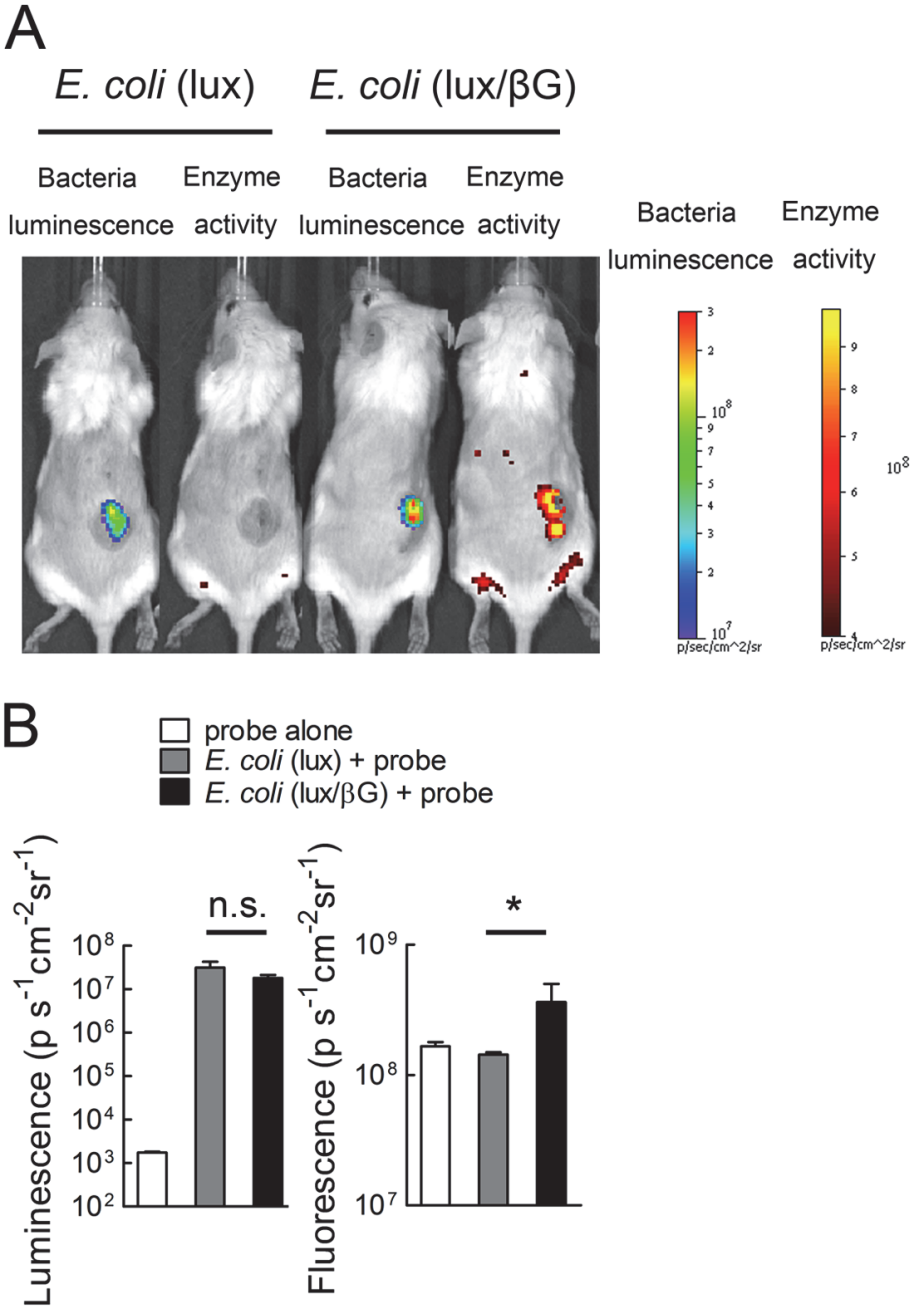
*In vivo* beta-glucuronidase activity of tumors treated with *E. coli* (lux/βG). (A) NOD/SCID mice bearing HCT116 tumors were i.v injected with 4 x 10^7^ c.f.u. *E. coli* (lux) or *E. coli* (lux/βG). After 4 days, 500 μg FDGlcU was i.v. injected into the mice. After 30 min, the luminescence (representing bacterial numbers) and fluorescence (representing beta-glucuronidase activity) in tumors were detected on an IVIS Spectrum (n = 3). (B) The mean luminescence and fluorescence intensities in tumors are shown. Unit is photons sec^-1^cm^-2^sr^-1^. Bars, s.d. * P < 0.05.

To investigate if the similar anticancer activity observed for treatment with CPT-11 and either *E. coli* (lux/βG) or *E. coli* (lux) could be caused by *in vivo* induction of endogenous beta-glucuronidase expression in *E. coli* (lux) or by *in vivo* loss of beta-glucuronidase expression in *E. coli* (lux/βG), we i.v. injected *E. coli* (lux/βG) or *E. coli* (lux) into mice bearing established HCT116 tumors and then i.v. injected a fluorescence glucuronide probe, FDGlcU, to measure *in vivo* beta-glucuronidase activity (Fig. 7A). FDGlcU does not emit fluorescence unless the glucuronide group is enzymatically removed. *E. coli* (lux/βG) displayed similar luminescence emission but higher fluorescence emission than *E. coli* (lux) (Fig. 7B), indicating that similar numbers of bacteria were in tumors but that *E. coli* (lux/βG) expressed significantly more beta-glucuronidase activity in tumors than did *E. coli* (lux). We conclude that *E. coli* (lux/βG) and *E. coli* (lux) displayed the expected high and low beta-glucuronidase activity in tumors.

### Efficient glucuronide hydrolysis by adenoviral expression of beta-glucuronidase

**Fig 8:**
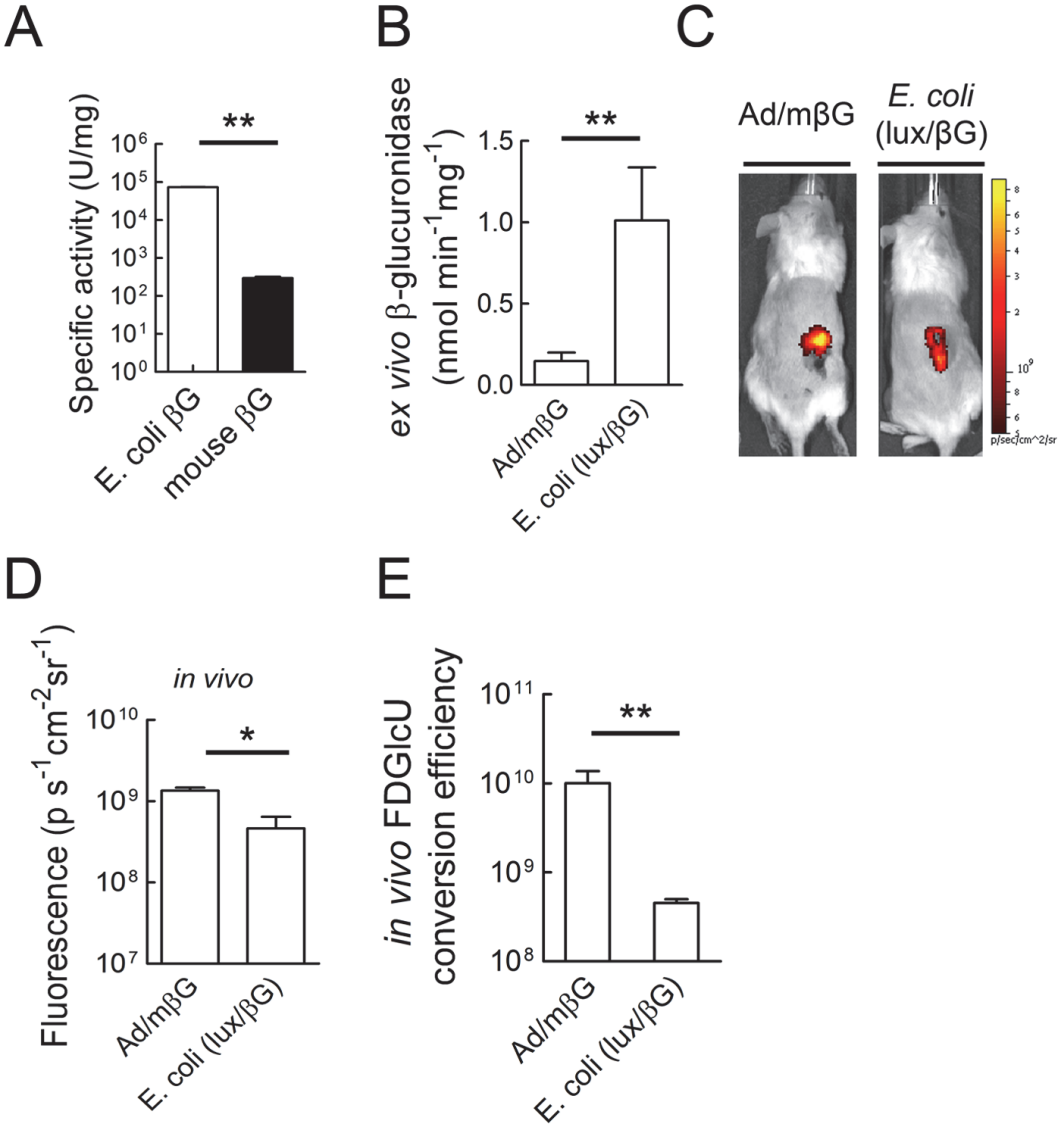
Comparison of *in vivo* beta-glucuronidase activity of tumors treated with Ad/mβG or *E. coli* (lux/βG). (A) The specific activities of *E. coli* and mouse beta-glucuronidase for hydrolysis of SN-38G at pH 7 were determined (n = 3). Bars, s.d. (B) Mice bearing HCT116 tumor were treated with 10^9^ p.f.u. Ad/mβG intratumorally on two consecutive days or 4 x 10^7^ c.f.u. *E. coli* (lux) or *E. coli* (lux/βG) via i.v. injection. After 4 days, 50 μl homogenized tumors lysates were incubated with 50 μl 500 μM 4-MUG to measure beta-glucuronidase activity. Results represent mean values from three mice. Bars, s.d. (C) Mice bearing HCT116 tumors were treated intratumorally with Ad/mβG or i.v. injected with *E. coli* (lux/βG). On day 4, 500 μg FDGlcU was i.v. injected into each mouse (n = 3). The fluorescence signal represents the *in vivo* beta-glucuronidase activity. (D) The mean fluorescence in tumors after i.v injection of FDGlcU in mice previously treated i.v. with *E. coli* (lux/βG) or intratumorally with Ad/mβG was quantified. (n = 3). (E) The relative *in vivo* FDGlcU conversion efficiency was calculated by dividing the *in vivo* FDGlcU fluorescence in tumors by the beta-glucuronidase activity in tumor lysates (n = 3). * P < 0.05; ** P < 0.005; n.s. no significant difference.

We previously showed that intratumoral injection of Ad/mβG, which allows expression of murine beta-glucuronidase as a membrane-anchored form on the surface of infected cells, can enhance the anti-tumor activity of CPT-11 [47]. Therefore, we used adenovirus therapy as a control to identify possible mechanisms that led to *E. coli* therapy failure. To investigate differences between bacterial and adenoviral beta-glucuronidase therapy, we first compared the enzymatic activities of *E. coli* beta-glucuronidase (produced in *E. coli* (lux/βG) and murine beta-glucuronidase (produced by Ad/mβG). *E. coli* beta-glucuronidase hydrolyzed the glucuronidase substrate 4-Nitrophenyl β-D-glucopyranoside (pNPG) faster than did murine beta-glucuronidase at pH 7 [36]. Similarly, we found that *E. coli* beta-glucuronidase displayed about 250 fold greater enzymatic activity for the hydrolysis of SN-38G compared to murine beta-glucuronidase at pH 7 (Fig. 8A). Next, we measured the relative beta-glucuronidase activity in tumor homogenates prepared from mice treated by i.v. injection of *E. coli* (lux/βG) or intratumoral injection of Ad/mβG. Again, the total beta-glucuronidase activity in tumors was significantly greater in mice treated with *E. coli* (lux/βG) than those treated with Ad/mβG (Fig. 8B). These results show that systemic administration of *E. coli* (lux/βG) can deliver more beta-glucuronidase activity to tumors as compared to direct injection of tumors with Ad/mβG.

To assess the functional accessibility of beta-glucuronidase to systemically administered drugs, we i.v. injected the fluorescent glucuronide probe FDGlcU and then imaging the fluorescence intensity in tumors of mice treated i.v. with *E. coli* (lux/βG) or i.t. with Ad/mβG. Fluorescence signals were apparent in the tumors of mice receiving either treatment (Fig. 8C), but mice treated with Ad/mβG exhibited significantly more fluorescence in tumors as compared to the mice treated with *E. coli* (lux/βG) (Fig. 8D). A relative *in vivo* FDGlcU conversion efficiency was calculated by dividing the FDGlcU tumor fluorescence (from Fig. 8D) by the beta-glucuronidase activity in tumor lysates (from Fig. 8B). Tumors injected with Ad/mβG displayed about 22 fold greater FDGlcU conversion efficiency than tumors treated with *E. coli* (lux/βG) (Fig. 8E). We conclude that although treatment of mice with *E. coli* (lux/βG) produced more beta-glucuronidase activity in tumors, the beta-glucuronidase activity generated by Ad/mβG can more effectively hydrolyze a systemically administered glucuronide probe in the tumors.

### Beta-glucuronidase distribution in tumors may affect hydrolysis of a systemically administered glucuronide probe

**Fig 9:**
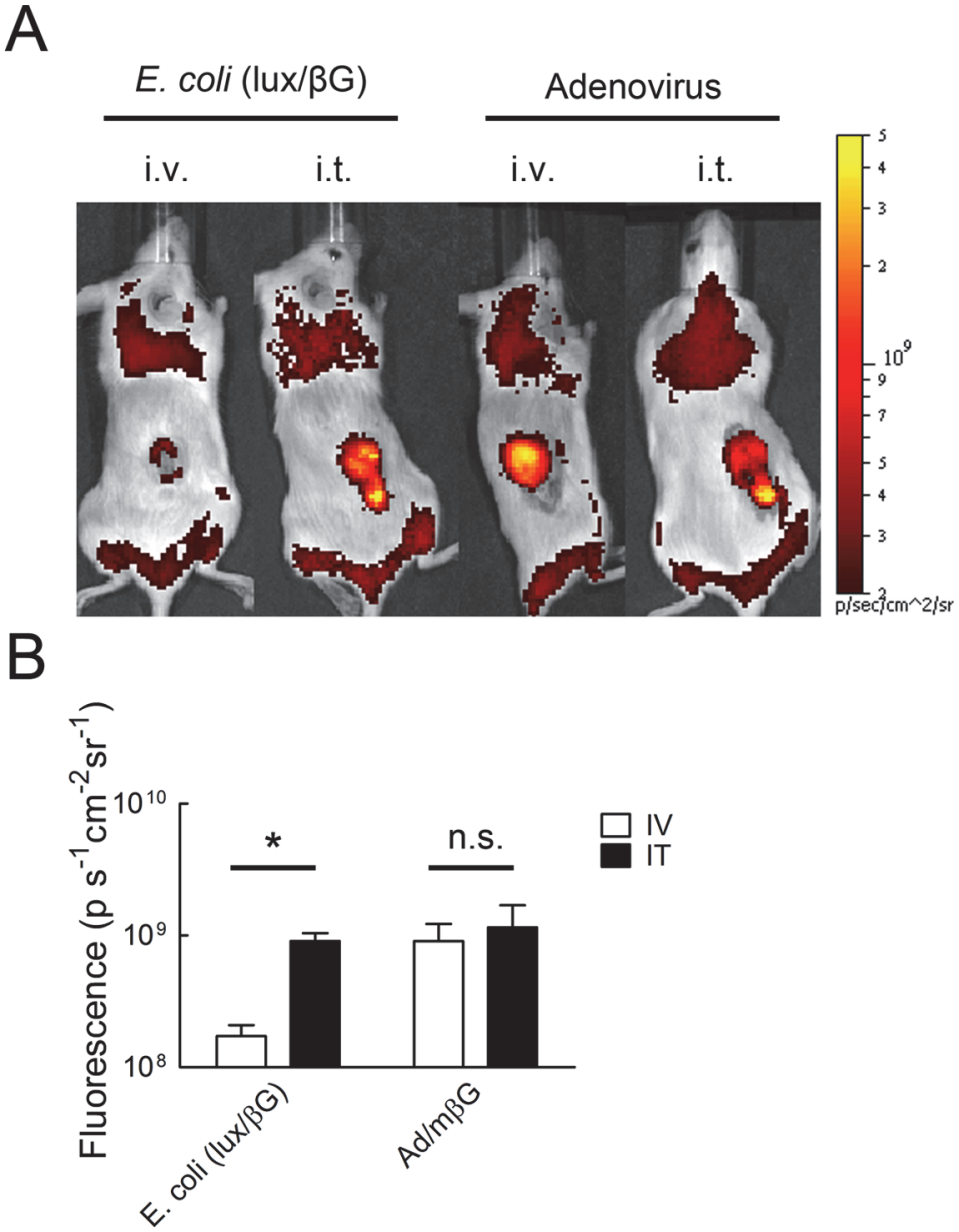
Intratumoral injection of FDGlcU in mice treated with *E. coli* (lux/βG) enhances glucuronide hydrolysis. (A) NOD/SCID mice bearing HCT116 tumors were i.v. injected with 4 x 10^7^ c.f.u. *E. coli* (lux/βG) or intratumorally-injected with 10^9^ p.f.u. Ad/mβG on two consecutive days. On day 4, 500 μg FDGlcU was i.v. injected into mice or 5 μg FDGlcU was directly injected into tumors. After 30 min, the fluorescence of the tumors were detected on an IVIS Spectrum (n = 3). (B) The region of the interesting (ROI) of mice treated as in panel A were analyzed (n = 3). The Y-axis indicates the mean FDGlcU fluorescence intensity (n = 3). Bars, s.d. * P < 0.05; n.s. no significant difference.

The relatively ineffective hydrolysis of FDGlcU in tumors treated with *E. coli* (lux/βG) suggested that the chemical probe delivered via the tumor blood supply may not effectively contact beta-glucuronidase in the tumor. To test this idea, we compared the fluorescence in tumors after systemic (i.v.) or direct intratumoral (i.t.) injection of FDGlcU to mice previously treated with *E. coli* (lux/βG) or Ad/mβG. The dose of FDGlcU was decreased by 100 fold for intratumoral injection groups to prevent saturation of beta-glucuronidase in the tumors. While mice treated with Ad/mβG displayed similar fluorescence after either i.v. or i.t. injection of FDGlcU, the mice treated with *E. coli* (lux/βG) exhibited more tumor fluorescence after i.t. injection of FDGlcU (Fig. 9A). Mean tumor fluorescence from groups of three mice treated with *E. coli* (lux/βG) was significantly greater when FDGlcU was directly injected into tumors as opposed to after i.v, administration (Fig. 9B), suggesting that systemically administered FDGlcU was less able to come into contact with beta-glucuronidase in *E. coli* as opposed to beta-glucuronidase expressed in cancer cells after administration of Ad/mβG.

### Release of beta-glucuronidase from bacteria increases hydrolysis of a systemically administered glucuronide probe

**Fig 10:**
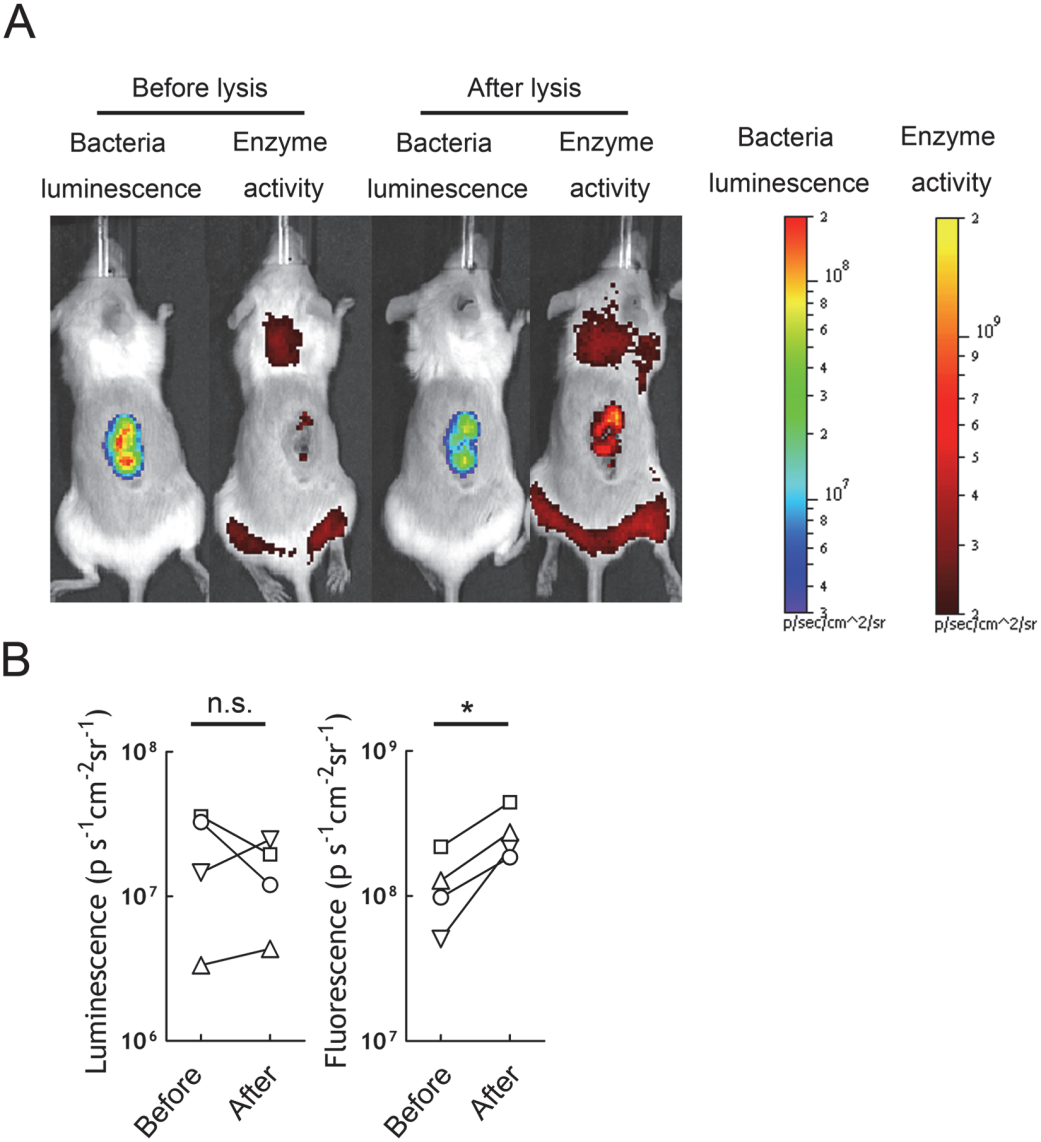
Lysing *E. coli* in tumors can increase glucuronide hydrolysis. (A) NOD/SCID mice bearing HCT116 tumors were i.v. treated with 4x 10^7^ c.f.u. *E. coli* (lux/βG). 500 μg FDGlcU was i.v. injected into mice and imaged on day 4 (before lysis reagent treatment). Two days later, the same mice were treated with 50 μl 4 mg/ml lysozyme and DNase I by intratumor injection to break the bacteria and release the enzyme into tumor tissue. After treatment, 500 μg FDGlcU was immediately given by i.v. injection. Mice were imaged (after lysis reagent treatment) 30 min later and (B) the ROI were analyzed (n = 4). * P < 0.05; n.s. no significant.

To investigate if the limited probe hydrolysis by poor contact between beta-glucuronidase in *E. coli* and FDGlcU could be reversed by generating soluble beta-glucuronidase in tumors, we i.v. injected *E. coli* (lux/βG) to allow tumor colonization and then directly injected a mixture of lysozyme/DNase I into tumors to break the bacteria cell wall. This approach was used to release beta-glucuronidase from the bacteria because the enzyme is too large (~280 kDa) to be efficiently secreted (results not shown). Mice were first i.v. injected with FDGlcU on day 4 to measure the fluorescence generated by hydrolysis of FDGlcU by intact bacteria in the tumors. After 48 h, the lysozyme/DNase I mixture was injected into tumors and then FDGlcU was again i.v. injected and tumor fluorescence was imaged. Imaging of bacterial luminescence showed the number of bacteria in tumors on days 4 and 6 were not significantly different (Fig. 10A and B). By contrast, i.t. injection of lysis solution resulted in stronger tumor fluorescence in images (Fig. 10A right) and significantly increased (p = 0.01) tumor fluorescence from FDGlcU hydrolysis (Fig. 10B). We conclude that release of beta-glucuronidase from *E. coli* in tumors can enhance the effective hydrolysis of a systemically-administered glucuronide compound.

## Discussion

Methods to enhance the selectivity and efficacy of cancer chemotherapy are needed to improve patient outcome. Bacteria that have been modified to express enzymes for tumor-selective activation of proactive anticancer agents have been investigated as a promising approach to achieve more selective cancer therapy [8,48]. Here we investigated if the anticancer activity of the clinically used anticancer drug CPT-11 could be enhanced by systematic administration of *E. coli* that were engineered to express *E. coli* beta-glucuronidase. The rationale for this approach is that high levels of SN-38G, a non-toxic glucuronide metabolite, are present in the blood of patients that receive CPT-11 [34,35]. Conversion of SN-38G to the highly potent topoisomerase I poison SN38 in tumors might enhance the activity of CPT-11 therapy. Indeed, we previously demonstrated that expression of murine beta-glucuronidase on cancer cells can significantly enhance the anticancer activity of CPT-11 in mice bearing human tumor xenografts [4,36,37]. These results are encouraging because SN-38G/SN-38 ratios in human serum are greater than those found in mice receiving CPT-11, suggesting that intratumoral conversion of SN-38G to SN-38 may produce superior antitumor activity in humans. We predicted that *E. coli* engineered to constitutively express *E. coli* beta-glucuronidase would more effectively enhance CPT-11 anticancer activity because *E. coli* beta-glucuronidase is about one hundred times more active than murine beta-glucuronidase. Indeed, systemically-administration of engineered *E. coli* generated significantly more beta-glucuronidase activity in tumors as compared to direct tumor injection of an adenoviral vector that expressed murine beta-glucuronidase on transfected cells. However, we found that microbially-delivered *E. coli* beta-glucuronidase did not significantly enhance the antitumor activity of CPT-11. We identified two impediments that appear to reduce the effectiveness of BDEPT using beta-glucuronidase: 1) slow uptake of glucuronide compounds into *E. coli* may reduce the rate of prodrug hydrolysis and 2) the preferential accumulation of *E. coli* in necrotic regions of tumors may hamper efficient contact of systematically administered drugs with beta-glucuronidase. Our study suggests that both the cellular and spatial distribution of beta-glucuronidase, and possibly other therapeutic enzymes, might be an important barrier for effective bacteria-based prodrug cancer therapy.

*E. coli* was selected in our study to deliver beta-glucuronidase to tumors. There are two major advantages of using *E. coli* as a delivery vehicle for prodrug activation. First, *E. coli* can selectively colonize solid tumors, providing high tumor/normal tissue colonization ratios of around 10,000 fold [17,18,38]. In agreement with these studies, we observed tumor/liver and tumor/spleen bacterial ratios of 9,700 and 23,000 at eight days after i.v. injection of *E. coli* to mice. By contrast, *Salmonella* provides tumor/liver and tumor/spleen bacterial ratios of 100~1000 [13,14]. Likewise, tumor selective antibodies typically achieve tumor/liver ratios of 1.4~7 in mice [21–24]. The high selectivity of *E. coli* for tumors may help reduce prodrug activation in normal tissues. Second, unlike liposomes, antibodies or replication deficient viruses, bacteria can proliferate in tumors [49–51]. The long-term colonization of tumors may allow multiple rounds of prodrug administration after a single injection of *E. coli*. These advantages have been recognized as indicated by the increasing number of papers investigating *E. coli* for tumor imaging and tumor therapy [15,17,19,20,38,52]. Of note, the strain of *E. coli* used in our study (DH5α) does not express enterotoxins or lipopolysaccharides and is considered to be non-pathogenic [53]. We used immune-deficient mice in most studies to investigate tumor therapy in human xenografts, but *E. coli* were also able to accumulate in murine tumors in immune-competent mice with similar toxicity as observed in immune deficient mice, suggesting that *E. coli* (DH5α) may be a suitable delivery vehicle for clinical applications.

Beta-glucuronidase is normally expressed at low levels in *E. coli* [46]. Thus, beta-glucuronidase levels were undetectable in wild-type *E. coli*. We constitutively expressed *E. coli* beta-glucuronidase in *E. coli* (lux/βG). These bacteria expressed about 60 ng beta-glucuronidase per 10^7^ c.f.u. of bacteria and could convert cytotoxic concentrations of SN-38 from SN-38G. Because *E. coli* beta-glucuronidase can hydrolyze SN-38G to SN-38 about 250-fold faster than murine beta-glucuronidase (Fig. 8A), we anticipated that bacterial-mediated delivery of *E. coli* beta-glucuronidase to tumors would greatly enhance the antitumor activity of CPT-11 based on our previous studies in which we showed that ectopic expression or viral-mediated expression of murine beta-glucuronidase on tumor cells resulted in intratumoral conversion of SN-38G to SN-38, thereby increasing the antitumor activity of CPT-11 in mouse models of human cancer [4,36,37]. Indeed, significantly more beta-glucuronidase activity accumulated in tumors of mice treated with *E. coli* (lux/βG) as compared to mice treated with Ad/mβG (Fig. 8B). However, microbally-expressed *E. coli* beta-glucuronidase did not increase CPT-11 antitumor activity beyond that achievable by treating tumors with wild-type *E. coli* (Fig. 6). This correlates with our observation that a systemically-administered glucuronide fluorescent probe was more effectively hydrolyzed in tumors treated with Ad/mβG than in tumors colonized by *E. coli* (lux/βG) (Fig. 8E).

Ad/mβG and *E. coli* (lux/βG) therapy differ in several important facets as indicated in Table 2. First, the enzyme distribution in the tumor microenvironment is different. Adenoviruses only infect live cells whereas *E. coli* preferred to colonize the margins between necrotic regions and live cells (Fig. 5A) [4]. Ad/mβG infected cells may therefore more easily interact than *E. coli* (lux/βG) with systemically-administered drugs. Second, the enzyme location in the cells differs. Ad/mβG was designed to express beta-glucuronidase on the cell membrane of infected cells whereas *E. coli* (lux/βG) expresses beta-glucuronidase in the periplasmic space of the bacteria. Glucuronide drugs can directly interact with membrane-anchored beta-glucuronidase but require receptor-mediated transport into *E. coli* to interact with beta-glucuronidase [54]. Third, the enzymes are derived from different sources; Ad/mβG expresses murine beta-glucuronidase whereas *E. coli* (lux/βG) expresses *E. coli* beta-glucuronidase. The enzymatic activity of *E. coli* beta-glucuronidase is much greater than mouse beta-glucuronidase (Fig. 8A).

**Table 2:** Differences between Ad/mβG and *E. coli* (lux/βG).

Therapy	Ad/mβG	*E. coli* (lux/βG)
Tumor distribution	Live cells	Border between live cells and necrotic areas
Enzyme location	Cancer cell surface	*E. coli* periplasmic space
Enzyme source	Mouse beta-glucuronidase	*E. coli* beta-glucuronidase

We identified two impediments that may account for the relatively poor performance of *E. coli* (lux/βG) as compared to Ad/mβG. First, beta-glucuronidase is expressed intracellularly in *E. coli* (lux/βG) but is present on the surface of cells as a membrane anchored form after infection with Ad/mβG. Thus, glucuronide substrates must enter bacteria, presumably via a glucuronide transporter complex present on *E. coli* [54], to contact beta-glucuronidase present inside the bacteria. This step appeared to be rate-limiting as shown by the approximately 10.1 fold and 7.7 fold slower hydrolysis of 4-MUG and SN-38G, respectively, by the same amount of beta-glucuronidase in intact bacteria compared to bacterial lysates (Fig. 1D and E). No such barrier exists for membrane-anchored beta-glucuronidase, which may allow efficient hydrolysis of glucuronide substrates [55]. The second factor contributing to the poor performance of microbially-delivered beta-glucuronidase appears to be related to localization of bacteria in necrotic areas of the tumor, which may hinder contact of systemically administered drugs and bacteria. In agreement with Westphal *et al* [38], we observed that *E. coli* preferentially accumulated at the border between viable cancer cells and necrotic regions (Fig. 5). *Salmonella* and *Shigella* have also been observed to colonize in the necrotic areas of tumors [38], suggesting that this may be a general phenomenon. The necrotic areas in tumors are distant from blood vessels and display high interstitial fluid pressure [56–58] which may constitute a physiological barrier for drug delivery in solid tumors [59,60]. The high interstitial fluid pressure might impede drug diffusion to areas of bacterial tumor colonization. On the other hand, adenoviruses infect live cells which are located in the outer region of tumor tissue. Systemically-administered drugs might therefore more easily interact with beta-glucuronidase on Ad/mβG infected cancer cells. In agreement with these ideas, direct intratumoral injection of a glucuronide probe to tumors previously colonized with *E. coli* (lux/βG) significantly increased probe hydrolysis (Fig. 9B), consistent with drug distribution as a limiting factor in conversion of the glucuronide probe to fluorescent product in the tumors. Likewise, lysing *E. coli* (lux/βG) in the tumor by intratumoral injection of lysozyme and DNase I to release beta-glucuronidase also significantly increased hydrolysis of the glucuronide probe (Fig. 10B). Taken together, these results support the idea that bacterial distribution and cellular enzyme location are important factors for BDEPT of glucuronide prodrugs. Future studies that measure activated drug concentrations in tumors and serum will be important to confirm these results.

Besides *E. coli*, other bacteria such as *Salmonella* and *Shigella* also prefer to colonize in the necrotic regions of tumors [38]. Thus, designing secreted forms of therapeutic proteins [61–67] or inducing bacteria lysis to release intracellular proteins from the bacteria [66] might represent general approaches to maximize cancer cell and drug contact by allowing diffusion of the proteins more evenly in tumor tissue as well as by removing bacterial barriers to allow unhindered contact between enzymes and drug molecules.
